# The impact of trade with the United States on electric loads in Mexico

**DOI:** 10.1016/j.heliyon.2018.e00717

**Published:** 2018-08-02

**Authors:** Marc H. Vatter, Daniel F. Suurkask

**Affiliations:** Elevation Direct, 3753 Howard Hughes Pkwy, Las Vegas, NV 89169, USA^1^

**Keywords:** Economics, Business, Energy

## Abstract

We quantify the relationship between trade with the U.S. and electric loads in Mexico. Exports to the U.S. are highly statistically significant in explaining energy loads and significant in explaining peak loads, both in the presence and the absence of a deterministic trend in loads. These results support the hypothesis that trade impacts industrial load disproportionately. We conclude that a failure to renegotiate NAFTA is an important regulatory risk for buyers and sellers of electricity in Mexico. We offer an approach to adjusting forecasts accordingly.

## 1 Introduction

Renegotiation of the North American Free Trade Agreement (NAFTA) raises innumerable issues with regard to specific goods and services. We focus here on the impact it may have on consumption of electricity in Mexico. Electric load growth in Mexico has exceeded economic growth, which is not the global norm. Industrial loads have accounted for a large share of that load growth. A prospective decline in exports of manufactured goods to the U.S. would affect planning for the supply of electricity in Mexico, and we develop some insight and guidance here for forecasters and planners.

The electric power sector in Mexico has recently undergone reform based on precedents established in other countries. A formerly vertically integrated government monopoly, Comisión Federal de Electridad (CFE), has been restructured into transmission, distribution, and generation subsidiaries, six of which compete with one another and other entrants to supply the wholesale market. Spot and forward markets are operated by Centro Nacional de Control de Energía (CENACE). The reform has created a good deal of opportunity for development and need for planning and analysis, but the specter of a failure of NAFTA introduces an element of risk.

In particular, in modeling the restructured electric power market in Mexico, would forecasts of energy and peak loads be substantially affected? Should scenarios be “run” in which Mexico experiences lower exports to the U.S.? How should load forecasts be adapted to account for a possible decline in Mexican exports to the U.S.? How should overall Mexican GDP be adjusted in forecast models in relation to exports to the U.S.?

To help answer these questions, we estimate the effects of Mexico's exports to the U.S. on energy and peak loads by Mexican electrical region (simplified to “region” hereafter), controlling for GDP and a trend toward energy efficiency.

Related academic literature covers a variety of topics. Ozawa et al. (2002) examine the relationship between energy use and CO_2_ emissions in Mexico's iron and steel industries. Destek (2016) finds that negative shocks to renewable energy consumption cause positive shocks to GDP in Mexico. Neme Castillo et al. (2015) find that energy consumption and industrial production and employment in Mexico are interdependent. Many articles discuss the effects of NAFTA. For example, Moreno-Brid et al. (2005) contrast surging non-oil exports with slower than expected overall economic growth and employment.

In Section 2, we provide a brief overview of trade and electric loads in Mexico, including key drivers. In Section 3, we present the sample data used in our analysis. Section 4 describes our econometric methods and models. Section 5 presents and discusses results, and we conclude in Section 6.

## 2 Background

NAFTA liberalized trade throughout North America and came into effect in 1994. From 1990 to 1994, Mexico imported more from the U.S. than it exported to the U.S., but it has run trade surpluses with the U.S. since then. The surpluses grew at an annual rate of 8.6% during 1997–2014 (World Bank, 2016).

The Mexican surpluses are also U.S. deficits, and President Trump has made an issue of this. NAFTA is being renegotiated, and the U.S. has repeatedly threatened to end the treaty if the three countries cannot agree on a revision. “…U.S. negotiators formally proposed that a new deal with Canada and Mexico favor American manufacturing and end after five years if all countries do not renew it.” (Pramuk, 2017).

Between 1997 and 2015, electric energy loads in Mexico grew at an average annual rate of 3.13% (Secretaría de Energía, 2016). We quantify the role played by growth of exports to the U.S. in the growth of electric loads and suggest a way for forecasters to model a scenario with slower growth of exports going forward.

An examination of the past few decades might lead one to surmise that Mexico is not capable of rapid economic growth, and that it will not realize its aspirations of developed country status. In fact, however, the country's economy has undergone a fundamental transformation during that time. Low end manufacturing of clothing, textiles, and simple assembly no longer dominates the industrial scene. It now constitutes only a part of a diversified industrial base that includes high end manufacturing. Mexico is now the world's seventh largest automobile manufacturer. Many of the leading carmakers have or soon will have facilities in Mexico (Kitroeff, 2016). The aerospace, medical device, and plastics industries have also experienced rapid growth.

Mexico has become a manufacturing powerhouse, in large part because of its access to the United States and comparatively low wages. Manufacturing now makes up about 18 percent of gross domestic product (Stratfor, 2015).

Heavy industry initially developed in northern Mexico, near the United States. Monterrey, in the Northeast region, is a major steel producer and has a very large manufacturing base. The northern states of Chihuahua and Baja California manufacture electronics.

More recently, high end and medium capital-intensive manufacturing have developed in central Mexico, in states such as Aguascalientes, Guanajuato, Queretaro, and San Luis Potosi, an area referred to as the “Bajio” (The corresponding electric region is Occidental.).

In the Oriental and (Yucatan) Peninsular regions, the petroleum and tourist industries are now complemented by low-end manufacturing of clothing and textiles, whose contribution to the economy continues to grow. Increasing wages in China have made these regions more competitive worldwide.

**Fig. 1:**
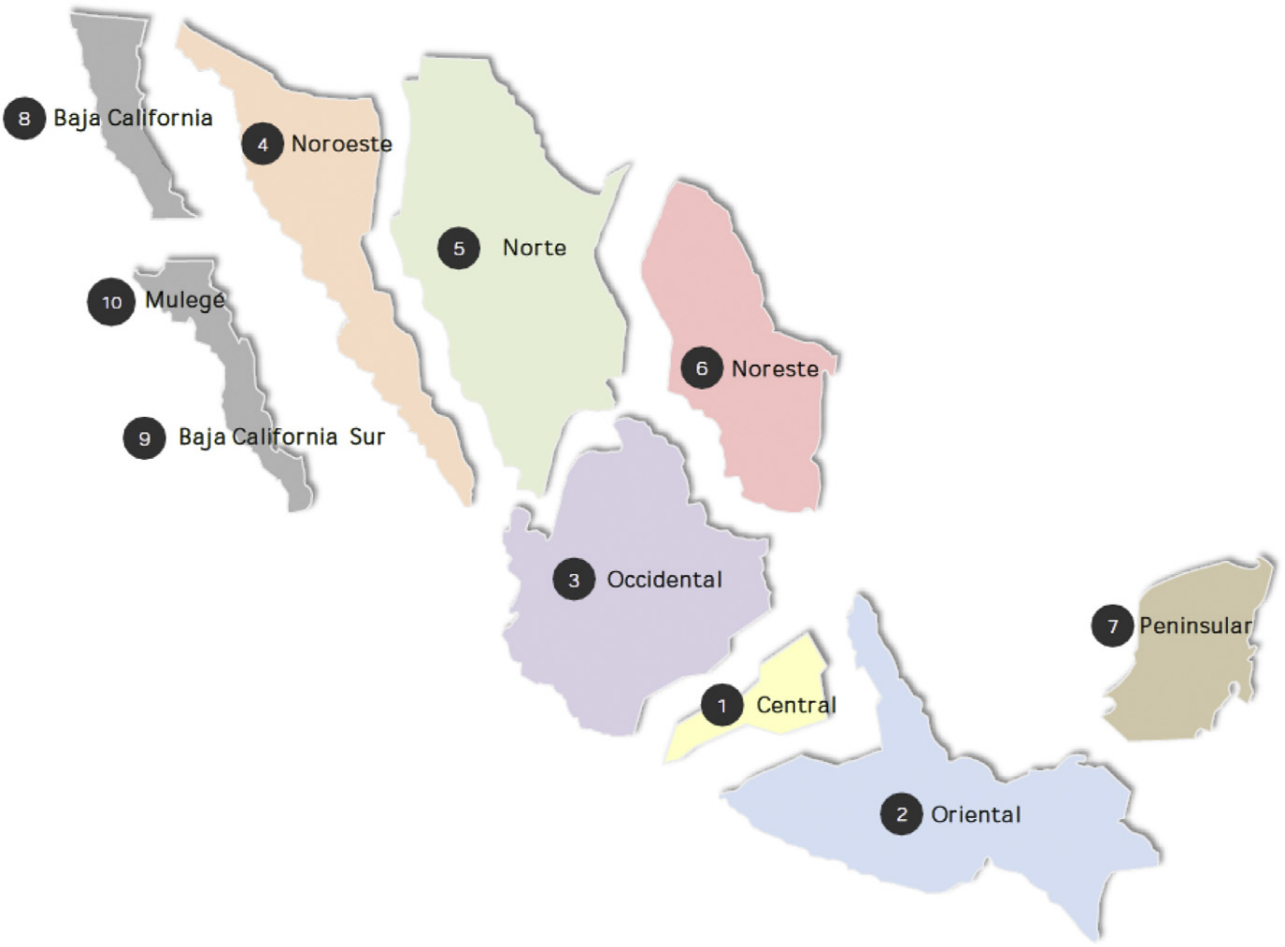
Mexican regions. Source: SENER, 2016; https://www.gob.mx/sener/acciones-y-programas/programa-de-desarrollo-del-sistema-electrico-nacional-33462, accessed June 13, 2018.

Fig. 1, below, shows a map of the electric control regions in Mexico, which we use as geographic categories in our analysis.

**Fig. 2:**
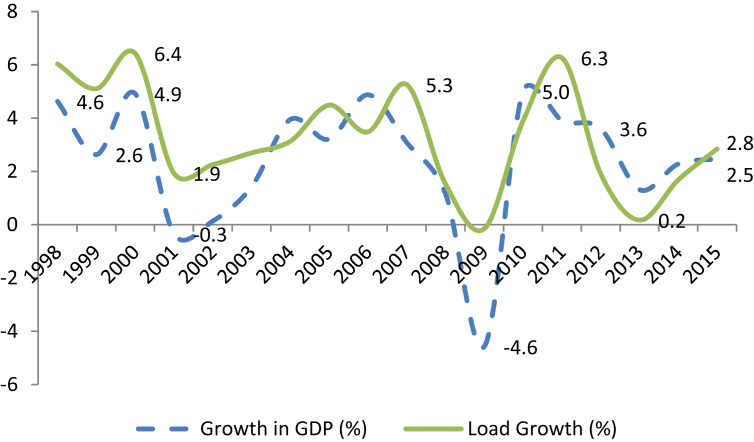
GDP & load growth, 1997–2015. Sources: Secretaría de Energía (SENER, 2018) and Organization for Economic Cooperation and Development (OECD, 2016). https://www.gob.mx/terminos, accessed June 29, 2018.

The economic transformation has had its effect on electric load growth and load shapes. Nationally, load growth averaged 3.1 percent from 1997 to 2015, and GDP grew at 2.4 percent. Of course, growth in GDP was especially slow during the Great Recession. Growth in both series was quite variable, as shown in Fig. 2.

The average load factor in the electric grid covering most of Mexico was 78% between 2010 and 2015 (Load factor is average load over peak load.). This results from growth in manufacturing under NAFTA. We also observe less use of air conditioning in Mexico than in an all-desert region because much of the population resides in and around Mexico City, which has a mild climate. In northern Mexico, temperatures are more extreme, and load factors are lower. Air conditioning drives peak loads in northern Mexico, and they occur in the summer. In Mexico City, the annual peak occurs around Christmastime, owing to decorative lighting, electric space heating, and the additional lighting requirements for illumination during the shorter winter days.

**Fig. 3:**
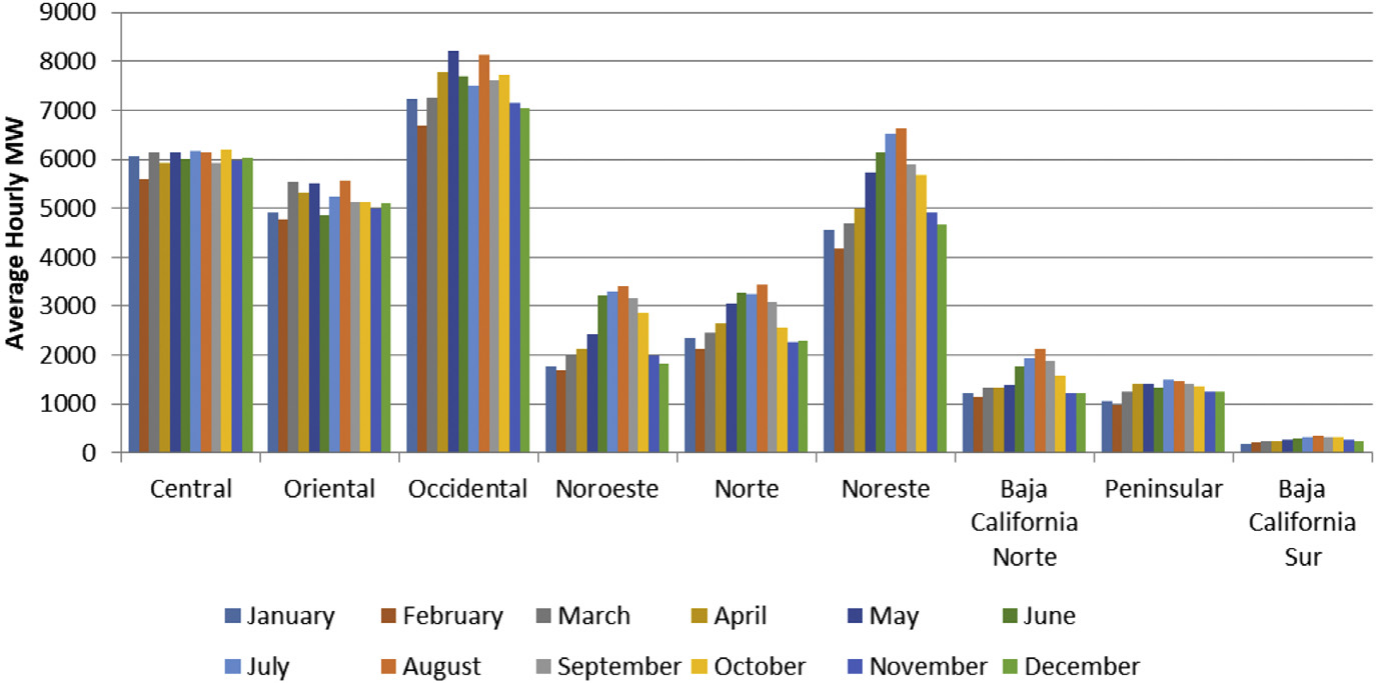
Monthly load by region. Source: (SENER, 2018), with modifications by authors. https://www.gob.mx/terminos, accessed June 29, 2018.

Fig. 3 shows monthly load by region for 2016. Central and Occidental, where loads are highest, have comparatively flat annual load profiles, owing to temperate weather year-round. The Baja California Norte, Noreste, Noroeste, and Norte loads exhibit sharper summer peaks.

## 3 Materials

We use historical load data from CFE (CFE, 2018) and the Secretaría de Energía (SENER, 2018). Historical data on Mexican trade and GDP come from the Organization for Economic Cooperation and Development (OECD, 2016) and the World Bank (World Bank, 2018). Table 1 shows the basic data used in our analysis. While Mexico has run a trade surplus with the United States, imports have exceeded exports overall.

**Table 1:** Data.^a^

	Baja Norte	Baja Sur	Central	Noreste	Noroeste	Norte	Occidental	Oriental	Peninsular	Mexico		
										Exports to U.S.	Trade Surplus	GDP
										$Million	$Million	$Billion
Gigawatt hours												
1997	6,739	1,039	37,059	26,208	11,900	12,006	33,947	26,524	4,523	102,787	−819	1,287
1998	6,954	1,063	38,859	28,571	12,100	12,970	36,178	28,260	4,923	112,232	−9,194	1,348
1999	7,674	1,130	40,711	30,293	12,519	13,624	38,385	29,231	5,201	130,904	−8,363	1,384
2000	8,641	1,200	43,080	32,464	13,046	14,698	40,955	30,835	5,711	158,561	−11,955	1,453
2001	8,927	1,231	44,515	32,958	13,463	15,404	40,682	31,040	6,123	147,634	−14,125	1,448
2002	8,827	1,231	45,335	34,040	13,120	15,856	41,774	32,259	6,331	149,165	−12,919	1,450
2003	9,334	1,282	46,313	34,406	13,649	16,178	43,262	33,021	6,746	155,637	−11,701	1,471
2004	9,723	1,380	47,573	35,660	14,259	16,742	44,633	33,556	7,156	178,908	−15,736	1,530
2005	9,926	1,505	49,459	36,952	15,134	17,768	47,159	35,081	7,359	197,708	−14,731	1,580
2006	10,516	1,662	50,863	38,459	15,583	18,253	48,646	36,286	7,872	225,937	−14,045	1,659
2007	11,272	1,841	51,953	41,068	16,616	19,416	51,603	38,322	8,353	237,947	−17,972	1,711
2008	11,418	2,068	52,430	41,824	16,690	19,347	52,405	39,107	8,854	248,483	−25,591	1,731
2009	11,100	2,121	52,158	41,470	16,997	19,437	52,179	39,096	9,216	197,626	−15,144	1,654
2010	10,991	2,152	54,227	43,442	17,339	20,403	55,602	40,098	9,206	251,495	−13,500	1,738
2011	11,426	2,302	55,108	47,379	19,251	22,116	60,066	42,447	9,735	287,497	−15,998	1,807
2012	12,020	2,353	54,866	47,776	20,097	22,484	61,665	43,835	9,938	301,349	−13,713	1,874
2013	11,996	2,386	53,891	47,581	20,466	22,679	61,974	44,224	10,300	316,007	−11,893	1,898
2014	12,598	2,460	53,228	48,559	21,089	23,150	63,540	44,901	10,635	336,419	−15,025	1,942
2015	13,122	2,546	53,649	50,114	21,642	23,734	65,220	46,587	11,617	328,094	−23,823	1,991
Megawatts												
1997	1,329	170	6,447	4,307	2,182	1,937	5,209	4,528	737			
1998	1,393	181	6,884	4,662	2,195	2,163	5,472	4,797	805			
1999	1,491	186	7,181	4,759	2,217	2,231	5,702	4,954	839			
2000	1,695	204	7,439	5,245	2,365	2,421	6,062	5,058	908			
2001	1,698	224	7,700	5,558	2,496	2,516	6,157	5,291	971			
2002	1,699	215	7,737	5,676	2,457	2,660	6,345	5,373	985			
2003	1,823	214	7,874	5,688	2,491	2,720	6,632	5,434	1,043			
2004	1,856	234	8,047	6,148	2,606	2,853	6,523	5,425	1,087			
2005	1,961	264	8,287	6,068	2,872	2,997	7,047	5,684	1,175			
2006	2,095	284	8,419	6,319	2,916	3,113	7,106	5,882	1,284			
2007	2,208	307	8,606	6,586	3,059	3,130	7,437	5,786	1,290			
2008	2,092	341	8,435	6,780	3,072	3,328	8,069	6,181	1,404			
2009	2,129	360	8,702	6,886	3,285	3,248	7,763	6,071	1,441			
2010	2,229	368	9,004	7,070	3,617	3,385	8,175	6,375	1,534			
2011	2,237	385	8,844	7,587	3,772	3,682	8,669	6,633	1,562			
2012	2,302	389	8,651	7,798	3,870	3,725	8,975	6,656	1,583			
2013	2,225	428	8,411	7,781	4,087	3,841	9,207	6,709	1,628			
2014	2,350	454	8,192	7,876	4,034	3,955	9,104	6,767	1,664			
2015	2,479	457	8,151	8,248	4,154	3,986	9,374	6,960	1,789			

a We used some of the same data in Vatter and Suurkask (2017).

## 4 Model

We regress annual percent changes in regional energy and peak loads on regional indicator variables and, depending on which of the four models estimated we are referring to, percent changes in exports to the U.S. and GDP, and a deterministic trend toward energy efficiency. An issue is controlling for variation in GDP when both exports and GDP are included in the model. Including both would normally give an estimate of the effect of variation in exports on load, holding GDP constant, effectively modeling the effects on loads of changes in the composition of GDP. However, we consider it preferable to allow GDP to vary, but only to the extent that it depends on exports. If exports to the U.S. increase, then aggregate demand increases. More people are employed, more raw materials are purchased, and more investment is done to satisfy increasing demand. Income and employment increase, leading to an increase in domestic GDP. Therefore, as a preliminary, we remove variation that depends on exports from the GDP variable before including it in a regression.

To do so, we must identify variation in GDP that depends on that in exports to the U.S. Exports may be endogenous to GDP because, for example, a rise in consumer confidence may increase demand for and production of all consumption goods, including those that would otherwise be exported, diverting them from foreign to domestic consumption. To find exogenous variation in exports to the U.S., we regress annual percent changes in exports on their lag, two lags of percent changes in the trade deficit, and two lags of percent changes in GDP.

(1) $$x_{t}^{US}-x_{t-1}^{US}=\alpha_{0}+\alpha_{x}^{US}\left(x_{t-1}^{US}-x_{t-2}^{US}\right)+\alpha_{d}^{1}\left(\ln\left[M_{t-1}-X_{t-1}\right]-\ln\left[M_{t-2}-X_{t-2}\right]\right)+\alpha_{d}^{2}\left(\ln\left[M_{t-2}-X_{t-2}\right]-\ln\left[M_{t-3}-X_{t-3}\right]\right)+\alpha_{g}^{1}\left(g_{t-1}-g_{t-2}\right)+\alpha_{g}^{2}\left(g_{t-2}-g_{t-3}\right)+\varepsilon_{t}^{x}$$

where $x_{t}^{US}$ is log exports to the U.S. in Year t, $M_{t}$ is imports, $X_{t}$ is exports (to all countries), $g_{t}$ is log GDP, and $\varepsilon_{t}^{x}$ is assumed to be spherical. Let $\Delta {\hat{x}}_{t}^{US}$ be the predicted value of $x_{t}^{US}-x_{t-1}^{US}$ from estimation of (1). We are looking for the part of exports to the U.S. that is exogenous to current GDP. We rely first on the fact that all of the instruments in (1) are lagged, and that the present does not cause the past. Second, many economic variables exhibit persistence of some kind, so we include lagged exports to the U.S. on the RHS. Third, exports are related to imports through currency markets; if, for example, imports rise, the peso may depreciate, causing exports to rise later. Third, lagged GDP affects current exports because, for example, an increase in aggregate demand may raise wages, and the price of exports, which will raise the total value of exports if demand for exports is inelastic, or lower it if demand for exports is elastic.

We regress the annual percent change in Mexican GDP on $\Delta {\hat{x}}_{t}^{US}$.

(2) $$g_{t}-g_{t-1}=\gamma_{0}+\gamma_{a}\Delta {\hat{x}}_{t}^{US}+\varepsilon_{t}^{g}={\hat{\gamma }}_{0}+{\hat{\gamma }}_{a}\Delta {\hat{x}}_{t}^{US}+e_{t}^{g}$$

where $e_{t}^{g}$, the residual shown on the second line, is statistically independent of exports. We then add the average percent change in GDP to this residual to form a variable that represents GDP and the variation therein that is independent of exports.

(3) $$g_{t}^{d}-g_{t-1}^{d}=\bar{\Delta g}+e_{t}^{g}$$

$g_{t}^{d}-g_{t-1}^{d}$ can be included in a regression with $\Delta {\hat{x}}_{t}^{US}$ so as to allow GDP to vary to the extent that it depends on exports, but to be otherwise held fixed as exports vary. Again, including both GDP and exports as regressors would hold GDP fixed as exports vary; (2) and (3) are necessary so that GDP may vary to the extent that it depends on exports. When the ordinary GDP variable is included as a regressor, GDP is held entirely fixed as exports vary, changes in exports imply a change in merely the composition of GDP, and, therefore, exports have no apparent effect on electric loads. We show that that is hardly the case when exports are allowed to affect GDP.

In considering models that predict electric loads, we begin with a simple model, Model I, in which energy and peak loads are interdependent, but follow constant rates of growth that are specific to each Mexican region.

(4) $$\begin{array}{l} q_{it}^{GWH}-q_{it-1}^{GWH}=\beta_{0}+\beta_{i}+\varepsilon_{it}\\ q_{it}^{MW}-q_{it-1}^{MW}=\delta_{0}+\delta_{i}+\mu_{it} \end{array}$$

$\varepsilon_{it}$ and $u_{it}$ may be heteroskedastic and inter-correlated. We estimate the equations in (4) simultaneously using seemingly unrelated regression, a consistent, asymptotically efficient, iterated maximum likelihood estimator, implemented using Stata's^®^ “sureg” command with the “isure” option. (These assumptions and techniques are not needed simply to calculate average rates of growth, as is done in (4), but they become useful as we add regressors.) The lower case variables represent natural logs. A model in differences in logs is a model of percentage rates of growth, and this differencing helps to minimize the possibility of spurious estimates of coefficients caused by non-stationarity in variables. $q_{it}^{GWH}$ is energy load in Region i in Year t, and ${\hat{q}}_{it}^{GWH}$is its predicted value using fixed effect estimators ${\hat{\beta }}_{X}$ of each corresponding $\beta_{X}$. $\beta_{0}$ is growth in energy load common to all regions, and the fixed, regional, effect on annual percent growth is captured in the term $\beta_{i}$ for Region i. (This is termed a “fixed” effect if $\beta_{i}$ is correlated with the regressors; only if not could it be modeled as a “random” effect.) $q_{it}^{MW}$ is annual peak load in Region i in Year t, and ${\hat{q}}_{it}^{MW}$ is its fixed effects estimator. $\delta_{0}$ is growth in peak load common to all regions, and the fixed, regional, effect in this equation is captured in the term $\delta_{i}$ for Region i. In general, $\beta_{i}\neq \delta_{i}$, so trends in growth may differ not only by region, but trend growth in consumption of energy may differ from that in peak load within a region.

i= (Baja California Sur, Central, Noreste, Noroeste, Norte, Occidental, Oriental, Peninsular). Baja California (Norte) is omitted to avoid the “dummy variable trap”, and Mulegé, which is very small, is not included in our analysis. We expect $\beta_{i}$ and $\delta_{i}$ to be relatively high in fast-growing areas, such as Peninsular, where growth has been driven by tourism in Cancun. We also expect $\beta_{i}-\delta_{i}$ to be relatively high in areas that have seen rapid industrialization because industrial loads tend to exhibit high load factors.

A random effects model could have improved on the fixed effects model if there had been many individuals in the panel, or time-invariant regressors specific to each region (See Kennedy, 2008, pp. 283-4.). However, there are only nine regions, and no regressors specific to those demarcations. Seemingly unrelated regression is thought to improve fixed effects models when “In practice, [the number of time-periods is] much larger than [the number of individuals]…” (See Baum, 2006, p. 237.) The sample of annual data covers nine regions over eighteen years, 1997–2015.

In Model II, we introduce percent changes in exports to the U.S.

(5) $$\begin{array}{l} q_{it}^{GWH}-q_{it-1}^{GWH}=\beta_{0}^{x}+\beta_{i}^{x}+\beta_{x}^{x}\Delta {\hat{x}}_{t}^{US}+\varepsilon_{it}^{x}\\ q_{it}^{MW}-q_{it-1}^{MW}=\delta_{0}^{x}+\delta_{0}^{x}+\delta_{x}^{x}\Delta {\hat{x}}_{t}^{US}+\mu_{it}^{x} \end{array}$$

where $\Delta {\hat{x}}_{t}^{US}$ is the predicted value from the first stage regression, (1). Since international trade includes manufactured goods, we might expect that $\beta_{x}^{x}>\delta_{x}^{x}$ due to the aforementioned tendency toward high load factors in industrial loads.

In Model III, we introduce percent changes in GDP that are independent of exports to the U.S.

(6) $$\begin{array}{l} q_{it}^{GWH}-q_{it-1}^{GWH}=\beta_{0}^{g}+\beta_{i}^{g}+\beta_{x}^{g}\Delta {\hat{x}}_{t}^{US}+\beta_{g}^{g}\left(g_{t}^{d}-g_{t-1}^{d}\right)+\varepsilon_{it}^{g}\\ q_{it}^{MW}-q_{it-1}^{MW}=\delta_{0}^{g}+\delta_{0}^{g}+\delta_{x}^{g}\Delta {\hat{x}}_{t}^{US}+\delta_{g}^{g}\left(g_{t}^{d}-g_{t-1}^{d}\right)+\mu_{it}^{g} \end{array}$$

Again, there is no variation in $g_{t}^{d}$ that depends on $x_{t}^{US}$, log exports to the U.S., so, as $x_{t}^{US}$ varies, GDP can vary to the extent that it depends on $x_{t}^{US}$; recall that $\Delta {\hat{x}}_{t}^{US}$ is the exogenous percent change in $x_{t}^{US}$. It may also be that $\beta_{g}^{g}\neq \delta_{g}^{g}$, so that growth in Mexican GDP may affect growth in peak load differently from how it affects growth in energy load.

It has been suggested that our regressions using the GDP variable calculated in (2) and (3) are equivalent to ordinary least squares (OLS). The coefficient on that GDP variable is, in fact, the same as in OLS, but the coefficient on (exogenously determined) exports to the U.S. is not. This is as intended: We do not wish to hold GDP entirely fixed as we allow variation in exports to cause variation in loads, as would be done in an OLS regression. Using matrix notation, an OLS regression would take the form

$$L=X\beta_{X}+G\beta_{G}+u$$

where L is the first difference in log load, G is the first difference in log GDP, X is the first difference in log exogenous exports to the U.S. and a constant term (a vector of 1's), and u is an error term. (We set aside the simultaneity of estimation of energy and capacity loads and regional effects because they only complicate the issue raised here.) By a corollary to the Frisch-Waugh Theorem (See Greene, 2003, p. 27), estimated coefficients on GDP, exports and the constant, respectively, are

$$\begin{array}{l} {\hat{\beta }}_{G}={\left(G^{\prime }M_{X}G\right)}^{-1}G^{\prime }M_{X}L\;M_{X}\equiv I-X{\left(X^{\prime }X\right)}^{-1}X^{\prime }\\ {\hat{\beta }}_{X}={\left(X^{\prime }M_{G}X\right)}^{-1}X^{\prime }M_{G}L\;M_{G}\equiv I-G{\left(G^{\prime }G\right)}^{-1}G^{\prime } \end{array}$$

where ${\hat{\beta }}_{X}$ includes both the coefficient on the export term and the constant.

Using (2) and (3), our GDP variable is $M_{X}G+\bar{G}$, where $\bar{G}$ is the sample mean of G, so our model differs from the OLS model:

$$L=X\beta_{X}^{VS}+\left(M_{X}G+\bar{G}\right)\beta_{G}^{VS}+u$$

Again by the Frisch-Waugh Theorem, the estimated coefficient on the GDP variable is

$${\hat{\beta }}_{G}^{VS}={\left(G^{\prime }M_{X}G\right)}^{-1}G^{\prime }M_{X}\left(L-\bar{G}{\hat{\beta }}_{G}^{VS}\right)={\left(G^{\prime }M_{X}G\right)}^{-1}G^{\prime }M_{X}L-{\left(G^{\prime }M_{X}G\right)}^{-1}G^{\prime }M_{X}\bar{G}{\hat{\beta }}_{G}^{VS}={\hat{\beta }}_{G}-{\left(G^{\prime }M_{X}G\right)}^{-1}G^{\prime }M_{X}\bar{G}{\hat{\beta }}_{G}^{VS}$$

Therefore,

$${\hat{\beta }}_{G}^{VS}=\frac{{\hat{\beta }}_{G}}{1+{\left(G^{\prime }M_{X}G\right)}^{-1}G^{\prime }M_{X}\bar{G}}$$

The (1 × 1) term ${\left(G^{\prime }M_{X}G\right)}^{-1}G^{\prime }M_{X}\bar{G}$ is the estimated coefficient on G in a regression of $\bar{G}$, a constant across observations, on G and X. X includes a vector of 1's, the estimated constant term will equal $\bar{G}$, and the estimated coefficient on G will be zero. Therefore, ${\hat{\beta }}_{G}^{VS}={\hat{\beta }}_{G}$.

However, a similar result does not obtain for ${\hat{\beta }}_{X}^{VS}$.

$${\hat{\beta }}_{X}^{VS}={\left(X^{\prime }M_{G}X\right)}^{-1}X^{\prime }M_{G}\left(L-\bar{G}{\hat{\beta }}_{G}\right)={\left(X^{\prime }M_{G}X\right)}^{-1}X^{\prime }M_{G}L-{\left(X^{\prime }M_{G}X\right)}^{-1}X^{\prime }M_{G}\bar{G}{\hat{\beta }}_{G}={\hat{\beta }}_{X}-{\left(X^{\prime }M_{G}X\right)}^{-1}X^{\prime }M_{G}\bar{G}{\hat{\beta }}_{G}$$

The term ${\left(X^{\prime }M_{G}X\right)}^{-1}X^{\prime }M_{G}\bar{G}{\hat{\beta }}_{G}$ is the coefficient on X in a regression of $\bar{G}{\hat{\beta }}_{G}$, a constant across observations, on G and X. X includes a vector of 1's, and the coefficient on X will not, in general, be zero, so, generally, ${\hat{\beta }}_{X}^{VS}\neq {\hat{\beta }}_{X}$. Our model is not equivalent to OLS. The inclusion of regional indicator variables, as in (6) and (7), and a trend line, as in (7), does not change this result, nor does the simultaneity of estimation of energy and peak loads.

In Model IV, we introduce a deterministic trend toward energy efficiency (See SENER, 2014, for descriptions of energy efficiency programs.).

(7) $$\begin{array}{l} q_{it}^{GWH}-q_{it-1}^{GWH}=\beta_{0}^{t}+\beta_{i}^{t}+\beta_{x}^{t}\Delta {\hat{x}}_{t}^{US}+\beta_{g}^{t}\left(g_{t}^{d}-g_{t-1}^{d}\right)+\beta t+\varepsilon_{it}^{t}\\ q_{it}^{MW}-q_{it-1}^{MW}=\delta_{0}^{t}+\delta_{0}^{t}+\delta_{x}^{t}\Delta {\hat{x}}_{t}^{US}+\delta_{g}^{t}\left(g_{t}^{d}-g_{t-1}^{d}\right)+\delta t+\mu_{it}^{g} \end{array}$$

Time series and regression analysis are among the major methods used to forecast electric loads, according to Almeshaiei and Soltan (2011). Esteves et al. (2015) review the literature on long-term electric load forecasting. They find that statistical, regression, and econometric models are the dominant types studied and applied, as distinct from computer intelligence and alternative models. See p. 558. The regional effects capture the impact of unspecified variables that differ by region. The logging of variables relates percent, as opposed to absolute, changes, because the regions differ greatly in size. The differencing of variables insures stationarity and avoids spurious inference. If the Mexican economy is becoming more energy efficient, we expect the coefficients on t to be negative. Wing (2008, p. 24) attributes improving energy efficiency in the U.S. to technical progress within industries.

## 5 Results

Estimation of (1) gives.

$$\Delta {\hat{x}}_{t}^{US}=\underset{0\text{.}0103}{0\text{.}0481-}\underset{0\text{.}2356}{0\text{.}0841}\left(x_{t-1}^{US}-x_{t-2}^{US}\right)-\underset{0\text{.}0321}{0\text{.}3613}\left(\ln\left[M_{t-1}-X_{t-1}\right]-\ln\left[M_{t-2}-X_{t-2}\right]\right)+\underset{0\text{.}0143}{0\text{.}0369}\left(\ln\left[M_{t-2}-X_{t-2}\right]-\ln\left[M_{t-3}-X_{t-3}\right]\right)+\underset{1.0145}{1.3231}\left(g_{t-1}-g_{t-2}\right)-\underset{0\text{.}3958}{0\text{.}4207}\left(g_{t-2}-g_{t-3}\right)+\varepsilon_{t}^{x}$$

The adjusted $R^{2}$ is 0.6095, and the overall *F*-statistic is highly significant. We have chosen a sufficiently strong set of instruments without having chosen an excessively large number of lags.

Estimation of (2) gives

(8) $${\hat{g}}_{t}-{\hat{g}}_{t-1}=\underset{0\text{.}0018}{0\text{.}0115}+\underset{0\text{.}0175}{0\text{.}1947}\Delta {\hat{x}}_{t}^{US}$$

Both the constant and the coefficient are highly significant. The adjusted $R^{2}=0.4633$. Exports to the U.S. are an important component and driver of Mexican GDP.

Table 2 shows results from estimation of Models I-IV. The indicator variables correspond to $\beta_{i},\;\beta_{i}^{x},\;\beta_{i}^{g},\;\beta_{i}^{t}$ and $\delta_{i},\;\delta_{i}^{x},\;\delta_{i}^{g},\;\delta_{i}^{t}$ in Equations (4), (5), (6), and (7). The constants in Model I equal the growth in load in Baja California Norte (BCN), and the coefficients on the dummies equal regional deviations in growth from BCN. Energy loads in BCN grew at 3.7%, and peak loads there at 3.5%. Energy loads in Peninsular grew at 3.7+1.5=5.2%, and energy loads in Central grew at 3.7−1.6=2.1%. The $R^{2}=0.1001$ in the energy equation and 0.1162 in the peak load equation.

**Table 2:** Effects of exports to the U.S. on electric loads in Mexican regions.

		Model							
	Variable	I		II		III		IV	
		Coeff.	Std. Err.	Coeff.	Std. Err.	Coeff.	Std. Err.	Coeff.	Std. Err.
**GWH**									
Percent change	Exports to US			0.096	0.025***	0.096	0.023***	0.100	0.022***
	GDPd					0.553	0.111***	0.549	0.108***
	Year							−0.001	0.000**
Indicator	Baja Sur	0.013	0.009	0.017	0.009	0.017	0.008*	0.017	0.008*
	Central	−0.016	0.009	−0.016	0.009	−0.016	0.008*	−0.016	0.008*
	Noreste	−0.001	0.009	−0.002	0.009	−0.002	0.008	−0.002	0.008
	Noroeste	−0.004	0.009	0.001	0.009	0.001	0.008	0.001	0.008
	Norte	0.001	0.009	0.001	0.009	0.001	0.008	0.001	0.008
	Occidental	−0.001	0.009	0.000	0.009	0.000	0.008	0.000	0.008
	Oriental	−0.006	0.009	−0.004	0.009	−0.004	0.008	−0.004	0.008
	Peninsular	0.015	0.009	0.017	0.009	0.017	0.008*	0.017	0.008*
	Constant^a^	0.037	0.006***	0.028	0.006***	0.014	0.007*	2.685	0.817**
**MW**									
Percent change	Exports to US			0.063	0.032*	0.063	0.031*	0.068	0.030*
	GDPd					0.414	0.149**	0.408	0.144**
	Year							−0.002	0.001**
Indicator	Baja Sur	0.020	0.011	0.024	0.011*	0.024	0.011*	0.024	0.011*
	Central	−0.022	0.011*	−0.024	0.011*	−0.024	0.011*	−0.024	0.011*
	Noreste	0.001	0.011	0.003	0.011	0.003	0.011	0.003	0.011
	Noroeste	0.001	0.011	0.007	0.011	0.007	0.011	0.007	0.011
	Norte	0.005	0.011	0.004	0.011	0.004	0.011	0.004	0.011
	Occidental	−0.002	0.011	−0.001	0.011	−0.001	0.011	−0.001	0.011
	Oriental	−0.011	0.011	−0.011	0.011	−0.011	0.011	−0.011	0.011
	Peninsular	0.015	0.011	0.016	0.011	0.016	0.011	0.016	0.011
	Constant^a^	0.035	0.008***	0.028	0.008**	0.018	0.009*	3.511	1.091**

* Indicates that the coefficient is significant at the 95% level. ** Indicates that it is significant at the 99% level. *** Indicates that it is highly statistically significant.

a Applies to Baja California (Norte).

Model II introduces exports to the U.S. Exports to the U.S. are highly statistically significant in the energy equation, and significant, at the 95% level, in the equation predicting peak load. The $R^{2}=0.2001$ in the energy equation and 0.1649 in the peak load equation. In the absence of exports, regional effects explain peak load better than energy load, but, with exports included, the regressors in the energy equation have greater explanatory power. Exports to the U.S. drive loads through high load-factor, manufacturing customers. As a robustness check, we have alternately substituted log exports to the U.S. of machinery and electronics and of transportation for total exports to the U.S. in Model II. Exports of machinery and electronics are highly statistically significant in explaining both energy and peak load. Exports of transportation are significant at the 95% level in explaining energy load and at the 99% level in explaining peak load.

However, the domestic economy also determines electric loads. Model III introduces the GDP variable, from which variation related to exports to the U.S. has been removed. Variation in exports to the U.S. may still cause GDP to vary, but variation in GDP that is independent of exports to the U.S. is now controlled for. The GDP variable is highly statistically significant in the energy equation and significant at the 99% level in the peak load equation. Exports to the U.S. continue to be highly significant as a predictor of energy loads, and significant at the 95% level in the equation for peak load. Results are still consistent with exports to the U.S. consisting of manufactured goods produced by high load factor, industrial customers. The $R^{2}=0.3170$ in the energy equation and 0.2075 in the peak load equation. Domestic economic activity, as measured by the GDP variable from which dependence on exports to the U.S. has been removed, is also a stronger driver of energy than of peak load.

If we use a GDP variable from which variation dependent on exports has not been removed, the GDP variable has the same coefficients and standard errors, but exports, a component of GDP, lose all statistical significance.

Model IV includes a deterministic trend as a way to model increasing efficiency in the use of electric energy. As intended, its coefficients are negative. They are significant at the 99% level in the equations for energy and peak load. Exports to the U.S. are again highly significant in the equation predicting energy load and significant at the 95% level in the peak load equation. The GDP variable is highly statistically significant in the equation for energy load and significant at the 99% level in the peak load equation. The adjusted $R^{2}=0.3641$ in the energy equation and 0.2602 in the peak load equation. The trend adds explanatory power.

If we add the residuals from the energy equations in Models III and IV across regions, a Robinson test estimates the degree of fractional differencing required to render the sum of residuals from Model III level stationary to be $d=\underset{0\text{.}2075}{0\text{.}0436}$, and a Bartlett test assigns a *p*-value of 0.9927 to a null hypothesis that these residuals were generated by a white noise process, indicating that the model is well specified. A Robinson test estimates the degree of fractional differencing required to render the sum of residuals from Model IV level stationary to be $d=\underset{0.2035}{-0\text{.}3029}$, and a Bartlett test assigns a *p*-value of 0.6791 to a null hypothesis that these residuals were generated by a white noise process. Though the trend toward efficiency appears to have explanatory power, and Model IV is also well specified, it is likely somewhat less well specified than Model III.

In all four models, the largest difference in the regional effects between the equations for energy and peak load, $\beta_{i}-\delta_{i}$, occur in Central and Oriental. We attribute the differences in Central to a peak load shaving program there, and to the temperate climate and relatively flat load there, as shown in Fig. 3. The differences in Oriental, near Central America and west of the Yucatan Peninsula, we attribute to growth in light industry. Around the year 2000, low wages in China made it hard for Mexico to compete in world markets for light manufactured goods, but wages in China have risen considerably since then, and we anticipate an increase in load factors in both Oriental and Peninsular, but especially in Oriental.

## 6 Conclusion

In the introduction, we asked whether, in modeling the restructured electric power market in Mexico, forecasts of energy and peak loads would be substantially affected by exports to the U.S. Exports to the U.S. have a highly significant effect on energy loads and a significant effect on peak electric loads in Mexico, whether or not GDP and increasing efficiency in the use of electricity are controlled for. Both the model that includes GDP and the model that includes GDP and the trend are well specified, but the former is better specified, while the latter appears to have greater explanatory power. We conclude that renegotiation of NAFTA represents a significant regulatory risk to anyone buying or selling electricity in Mexico.

We asked in the introduction whether scenarios should be “run” in which Mexico experiences lower exports to the U.S. We recommend sensitivity analyses in which the effects of exports to the U.S. on electric loads, especially energy loads, are accounted for.

Finally, we asked how load forecasts should be adapted to account for a possible decline in Mexican exports to the U.S., and how overall Mexican GDP should be adjusted in forecast models in relation to those exports. A practical problem is that load forecasting models may not include exports separately as a predictor of loads, let alone exports to the U.S. specifically, though they typically do include GDP. Even the gold standard of Mexican load forecasting models, that used by SENER in its annual PRODESEN publication, uses GDP but not exports, to predict load (See SENER, 2016, pp. 57–60.).

A practical way to examine scenarios regarding exports, then, is to make the appropriate corresponding adjustment in GDP growth. In (10), 0.0240 is annual GDP growth during the sample period, 1997–2015, which included the Great Recession, and the other numeric values are from (8). To use this approach, one need not have an assumed forecast of growth in exports to the U.S., but an assumed ratio of growth in those exports to GDP growth; $\Delta x^{s}/\Delta g^{s}$. While GDP grew at 2.40% annually during the sample period, exports to the U.S. grew at 5.86%, so this ratio was 2.44.

(9) $$\Delta g^{s}=\Delta g^{b}-\left(0.0240-\frac{0.0115}{1-0.1947\frac{\Delta x^{s}}{\Delta g^{s}}}\right)\Delta g^{s}\equiv \text{scenario}\;\text{GDP}\;\text{growth}\;\text{rate}\Delta g^{b}\equiv \text{base}\;\text{case}\;\text{GDP}\;\text{growth}\;\text{rate}\Delta x^{s}\equiv \text{scenario}\;\text{growth}\;\text{rate}\;\text{of}\;\text{exports}\;\text{to}\;\text{the}\;\text{U.S.}$$

According to the World Bank, about 80% of Mexican exports go to the United States, so it is not too extreme to assume that a failure to renegotiate NAFTA would imply that exports to the U.S. would not continue to grow faster than GDP, but, rather, that exports to the U.S. and GDP grew at the same rate: 0.0115/(1−0.1947)=1.43%p.a., so one would lower expected GDP growth by 2.40%−1.43%=0.97% from what one would assume with NAFTA successfully renewed.

## Declarations

### Author contribution statement

Marc Vatter: Conceived and designed the experiments; Analyzed and interpreted the data; Contributed reagents, materials, analysis tools or data; Wrote the paper.

Daniel Suurkask: Analyzed and interpreted the data; Contributed reagents, materials, analysis tools or data; Wrote the paper.

### Funding statement

This research did not receive any specific grant from funding agencies in the public, commercial, or not-for-profit sectors.

### Competing interest statement

The authors declare no conflict of interest.

### Additional information

No additional information is available for this paper.
